# CAR requires Gadd45β to promote phenobarbital-induced mouse liver tumors in early stage

**DOI:** 10.3389/fonc.2023.1217847

**Published:** 2023-09-07

**Authors:** Takeshi Hori, Kosuke Yokobori, Rick Moore, Masahiko Negishi, Tatsuya Sueyoshi

**Affiliations:** ^1^ Pharmacogenetics Section, Reproductive and Developmental Biology Laboratory, National Institute of Environmental Health Sciences, National Institutes of Health, Research Triangle Park, NC, United States; ^2^ Department of Biomechanics, Institute of Biomaterials and Bioengineering (IBB), Tokyo Medical and Dental University (TMDU), Tokyo, Japan

**Keywords:** phenobarbital, chemical carcinogenesis, growth arrest and DNA-damageinducible 45 beta, TGFbeta signaling, irisin

## Abstract

Phenobarbital (PB) is an archetypal substance used as a mouse hepatocellular carcinoma (HCC) promotor in established experimental protocols. Our previous results showed CAR is the essential factor for PB induced HCC promotion. Subsequent studies suggested Gadd45β, which is induced by PB through CAR activation, is collaborating with CAR to repress TNF-α induced cell death. Here, we used Gadd45β null mice (Gadd45β KO) treated with N-diethylnitrosamine (DEN) at 5 weeks of age and kept the mice with PB supplemented drinking water from 7 to 57 weeks old. Compared with wild type mice, Gadd45β KO mice developed no HCC in the PB treated group. Increases in liver weight were more prominent in wild type mice than KO mice. Microarray analysis of mRNA derived from mouse livers found multiple genes specifically up or down regulated in wild type mice but not null mice in DEN + PB groups. Further qPCR analysis confirmed two genes, Tgfbr2 and irisin/Fndc5, were up-regulated in PB treated wild type mice but no significant increase was observed in Gadd45β KO mice. We focused on these two genes because previous reports showed that hepatic Irisin/Fndc5 expression was significantly higher in HCC patients and that irisin binds to TGF-β receptor complex that includes TGFBR2 subunit. Our results revealed irisin peptide in cell culture media increased the growth rate of mouse hepatocyte-derived AML12 cells. Microarray analysis revealed that irisin-regulated genes in AML12 cells showed a significant association with the genes in the TGF-β pathway. Expression of irisin/Fndc5 and Tgfbr2 induced growth of human HCC cell line HepG2. Thus, Gadd45β plays an indispensable role in mouse HCC development regulating the irisin/Fndc5 and Tgfbr2 genes.

## Introduction

According to current information from the American Cancer Society, liver cancer is a common neoplasm with more than 80000 diagnoses and causing more than 70000 deaths each year worldwide (1). It is one of the most common cancers and the third leading cause of cancer-related death in 2020 (2). Hepatocellular carcinoma (HCC) is the most frequent histologic type of liver cancer (3). Many rodent model systems were developed for studying HCC mechanisms and testing drug candidates. Among them, a two stage chemically induced HCC that employs N-diethylnitrosamine (DEN) as the genotoxic initiator and phenobarbital (PB) as the non genotoxic promoter has been used in numerous studies as an archetypal system (4).

PB is a barbiturate anticonvulsant and activates constitutive active/androstane receptor (CAR, NR1i3) (5). CAR, originally cloned as a homologue of orphan nuclear receptors, was found to be a transcription factor that activates drug/chemical metabolizing enzymes in the liver for detoxification in late ‘90 (6). Our group has revealed that CAR is an essential factor for liver tumor promotion by PB in the two stage experimental system (7). Although a substantial number of publications about molecular mechanisms about PB tumor promotion mediated by CAR were published since then, the details of that were poorly understood (8–16). One such study found that the expression of growth arrest and DNA-damage inducible 45 beta (Gadd45β), a molecule known to be involved in cell stress responses, was strongly induced in a CAR dependent manner by PB (13). Gadd45β, a member of Gadd45 family comprised of α, β, and γ, is induced by stress signals and mediates various protein interplay among protein factors that causes a wide variety of cellular responses including DNA repair, cell cycle control, senescence, and apoptosis (17, 18). For example, Gadd45β mediates its anti-apoptotic effect by promoting degradation of p53 *via* Src/PP2A/MDM2 pathway following arsenite treatment (19). Moreover, Gadd45β downregulates pro apoptotic JNK signaling by interacting with MAPK kinase 7 (MKK7) (20). In addition to the anti-tumorigenesis functions of Gadd45β, it also has pro-apoptotic activities suggesting that Gadd45β functions are contingent on the context of cell circumstances and interplay with other factors (17, 18, 21).

Among the three GADD45 molecules, only Gadd45β was strongly induced by PB, in a CAR dependent manner. Provocatively, Gadd45β protein interacts with CAR and serves as a scaffold protein for regulating the activity of mitogen-activated protein kinase (MAPK). Our recent results suggested that the CAR-Gadd45β complex reduces p38 MAPK phosphorylation and acts as a tumor suppressor (22). Moreover, p38 MAPK activation in mouse liver dramatically reduced tumor development (23) thus suggesting p38 MAPK activity mitigation by CAR-Gadd45β complex may be causing the observed HCC development. Furthermore, CAR-Gadd45β complex was found to suppress phosphorylation of JNK1 by MAPK kinase 7 (MKK7) and repress TNFα induced cell death. (13). Fittingly, in the absence of PB, hepatocyte-specific knock-out (KO) of either p38 MAPK or JNK1 signaling promotes DEN-initiated HCC in mice (24, 25). In other words, suppressing these kinase activities by PB or gene knockout may promote DEN-induced HCC in a similar mechanism. Thus, we hypothesized that MAPK suppression by CAR-Gadd45β complex is responsible for PB-induced HCC promotion. In a previous report, as a first step for evaluating Gadd45β in HCC development, we analyzed Gadd45β KO mice with short term treatment with PB for hepatocyte growth. Gadd45β KO mice treated with PB for 48 hr showed suppressed BrdUrd intake into hepatocytes (22). Consistent with our findings, Tian et al. showed that potent CAR activator 1,4-bis[2-(3,5-dichloropyridyloxy)]benzene (TCPOBOP), induced liver growth and that Gadd45β slowed this growth (26).

Our new finding through a cancer patient database analysis which suggests low survival rates associated with low *GADD45β* expression levels in the liver further prompted us to focus on Gadd45β. Although nongenotoxic chemicals including PB have been well established to activate CAR and eventually induce HCC in rodents, the same mode of action involving nongenotoxic chemicals and CAR was not observed in human HCC. However molecular pathways found in HCC development in rodents may partially overlap with that of humans despite CAR activation may not be involved. Hence, in this report, we compared tumorigenesis in WT and Gadd45β null mice from a C57BL/6J background for analyzing roles of Gadd45β in HCC development. Mice were treated with DEN at 5 weeks old and PB was administered from 7 weeks to 57 weeks of age. Obtained results showed the disappearance of PB-induced adenoma in Gadd45β null mice. HCC was observed in only WT mice; thus, these results suggest that Gadd45β plays critical roles in PB-induced tumor promotion. Among numerous PB-modulated liver genes differentially affected by the Gadd45β null mutation in microarray analysis, we focused on two genes, *Tgfbr2* and Irisin/*Fndc5*, for this report. Both genes were induced by PB in wild type but not in Gadd45β null mouse liver. Irisin, a peptide produced by the cleavage of Fndc5 protein, was originally found as a myokine induced by physical exercise (27) and now this peptide is known to be expressed in multiple organs including the liver (28). Interestingly, a recent report showed increased irisin expression in HCC patients. These genes were found to be involved in the regulation of cell growth and *TGFBR2* expression levels showed a strong association with the survival of liver cancer patients. Further studies about the genes affected by Gadd45β including *Tgfbr2* and *Fndc5* and their roles in the MAPK activity modulation by Gadd45β will contribute to mechanistic understandings of CAR-dependent HCC promotion in mice.

## Materials and methods

### Materials

Phenobarbital sodium salt, 1,4-bis[2-(3,5-dichloropyridyloxy)]benzene (TCPOBOP), anisomycin, and DEN were purchased from Sigma-Aldrich (St. Louis, MO, USA). Fugene 6 from Promega (Ann Arbor, MI) and Lipofectamine 3000 from Life Technologies (Grand Island, NY, USA) were used for cell transfection of plasmid DNA; TaqMan Gene Expression Assays (Thermo Fisher, Waltham, MA) for gene expression analyses used in this study were as follows: Akr1b7, Mm00477605_m1; Casp1, Mm00438023_m1; Cdh1, Mm01247357_m1; Cyp2b9, Mm00657910_m1; Cyp2b10, Mm00456591_m1; Cyp2b13, Mm00771172_g1; Gadd45β, Mm00435123_m1; Fndc5, Mm01181543_m1; Krt19, Mm00491980_m1; Nedd9, Mm01324843_m1; Sult1e1, Mm00499178_m1; Tgfbr2, Mm03024091_m1; and Mouse GAPDH Endogenous Control. Irisin peptide was a product of Raybiotech (Peachtree Corners, GA, USA). Expression plasmid for FNDC5/Irisin was constructed by amplifying cDNA fragment using mouse liver cDNA and cloned into pcDNA3.1 V5-His TOPO (Thermo Fisher). Human TGFBR2 expression vector (pCMV5B-TGFbeta receptor II) was a gift from Joan Massague and Jeff Wrana (Addgene plasmid #11766) (29),

### Animals and drug treatments

C57BL/6J (Catalog# JAX 000664) and Gadd45β KO (B6;129S6-Gadd45βtm1Flv/J) (Catalog# JAX 013101) were obtained from Jackson Laboratories (Bar Harbor, ME, USA) and were maintained at the National Institute of Environmental Health Sciences (NIEHS, USA). Mice received a single i.p. treatment with DEN (90mg/kg body weight) at 5 weeks of age as in previous reports (7, 30, 31). Two weeks after the DEN treatment, mice were placed on PB (0.5 g/l) *via* drinking water or normal water as a control for 50 weeks. The water bottle with or without PB was changed every week. Based on 5 ml water consumption per mouse for one day, we expected 0.875 g of PB/mouse for the study. At the end of the 50-week period, mice were euthanized by CO_2_ and livers were excised for mRNA analyses. Histopathological evaluation of livers for tumor induction in Gadd45β KO mice and wild-type (WT) C57BL/6J mice was performed to see changes in hepatocytes including hypertrophy, foci and tumors. All animal procedures were approved by the Animal Care and Use Committee at NIEHS, NIH, and performed humanely in accordance with Public Health Service Policy.

### Cell cultures and transient transfection

HepG2 (human hepatocellular carcinoma cell line) and AML12 (mouse hepatocyte cell line) were obtained from the American Type Culture Collection (ATCC). HepG2 and AML12 cells were cultured in Eagle’s minimum essential media and DMEM/F12 media, respectively, supplemented with 10% fetal bovine serum (FBS) and penicillin/streptomycin at 37°C with 5% CO_2_. HepG2 cells were transfected by Fndc5/Irisin and Tgfbr2 expression plasmids using Fugene 6 reagent according to the manufacturer’s instructions. FNDC5/Irisin and Tgfbr2 protein expressions were detected by western blotting analyses using 10% SDS page gels employing anti V5 tag antibody (Thermo Fisher) and anti Tgfbr2 monoclonal antibody (Santa Cruz Biotechnology), respectively. AML12 cells in 96 well plates (seeded 5000 cells per well) were treated with irisin (1 μg/ml or 2 μg/ml) at 24 hr. Seventy-two hr after transfection (HepG2) or irisin treatment (AML12), cell viability was determined using Cell Counting Kit-8 (Dojindo).

### Gene arrays

All tissue samples for gene expression analysis were obtained from non-tumorous regions of liver tissues. RNAs were purified from mouse livers using Trizol reagent (Thermo Fisher Scientific) and the RNeasy mini kit (QIAGEN, Valencia, CA, USA). Gene expression analysis was conducted using Agilent Whole Mouse Genome 4 × 44 multiplex format oligo arrays (014868) (Agilent Technologies, Palo Alto, CA, USA) following the Agilent 1-color microarray-based gene expression analysis protocol. Cy3 labeled cRNA was produced following the manufacturer’s protocol. Cy3 labeled cRNAs were fragmented and hybridized for 17 h in a rotating hybridization oven. Slides were scanned with an Agilent Scanner. Data were obtained using the Agilent Feature Extraction software (v12). The Agilent Feature Extraction Software performed error modeling, adjusting for additive and multiplicative noise. Genes were considered differentially expressed if they showed a fold-change of at least 1.5 with *p*-value < 0.05 tested by an ANOVA and Benjamini-Hochberg multiple test correction performed using OmicSoft Array Studio (Version 10) software. Ingenuity Pathway Analysis software (IPA, Qiagen) was utilized to search the canonical pathway, toxicity function, or upstream regulator. The pathways with Z-score >2 and <-2 were considered significantly activated and repressed pathways, respectively. Gene set enrichment analysis (GSEA, version 4.0.3) was performed with the oncogenic gene sets (H) from Molecular Signature Database (MSigDB, v7.1). Gene set with NOM *p*-value < 0.05 was considered as significantly enriched.

### Bioinformatics analysis

The transcriptome data as fragments per kilobase of transcript per million mapped reads upper quartile (FMPK-UQ) and clinical information of hepatocarcinoma patients in TCGA-LIHC was downloaded from the GDC data portal (https://portal.gdc.cancer.gov/). The hepatocarcinoma samples of 377 patients were downloaded. The patients were grouped as low and high GADD45B by FMPK score of GADD45B as lower than 40 (n=121) and greater than 85 (n=133), respectively. Subsequently, the patients in high GADD45B were grouped as low and high FNDC5 by FMPK score of FNDC5 as lower than 4 (n=55) and greater than 10 (n=57), respectively. The hepatocarcinoma patients were grouped as low and high FNDC5 by FMPK score of FNDC5 as lower than 1 (n=133) and greater than 6 (n=137), respectively. Kaplan-Meier survival curve with log-rank test was performed to compare differences in survival distributions by using Python lifelines (https://lifelines.readthedocs.io/en/latest/, version 0.27.1).

### Statistical analysis

Statistical analyses were performed using Graphpad Prism (version 8.3.0, Graphpad Software, La Jolla, CA). A Grubbs test was performed for the statistical outlier evaluation for the assessment of liver weight/body weight.

## Results

### Low GADD45B expression is associated with poorer overall survival in hepatocarcinoma patients in public database

**Figure 1:**
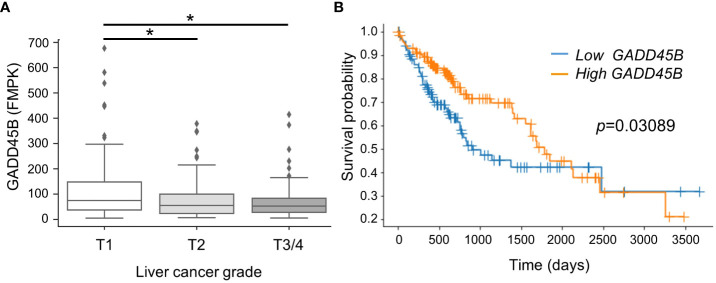
Lower expression of Gadd45β associated with poorer prognosis in human hepatocarcinoma. **(A)** Gadd45β expression levels in HCC patients. The box plot presented the median, first quartile, third quartile, and the vertical bars represent the 1.5*interquartile range (IQR). *P < 0.05 by one-way ANOVA followed by Sidak’s multiple comparisons. **(B)** Kaplan-Meier survival curves of patients with the high- (orange) or low- (blue) Gadd45β expression are displayed. Differences between survival curves were statistically analyzed by using the log-rank test.

In previous studies using the DEN/PB mouse HCC experimental system, our group found Gadd45β is induced in mouse liver through CAR activation by PB. Since Gadd45β has been considered to play an anti-tumor role in some cancers (18), our logical hypothesis was that this molecule is playing a key role in HCC tumorigenesis. Here we compared HCC patient survival rates between groups with low and high GADD45B expression. For this analysis, human HCC patients from the public domain (TCGA-LIHC) were grouped based on liver cancer stages. The data shows that GADD45B expression levels decreased according to liver cancer stage progression (**Figure 1A**). Strikingly, the Kaplan-Meier survival curve with log-rank test showed that the group with lower GADD45B expression has a poorer prognosis than the group with higher GADD45B expression, suggesting that GADD45B has antitumor functions in human HCC.

### Gadd45β is essential for PB promotion of HCC

**Figure 2:**
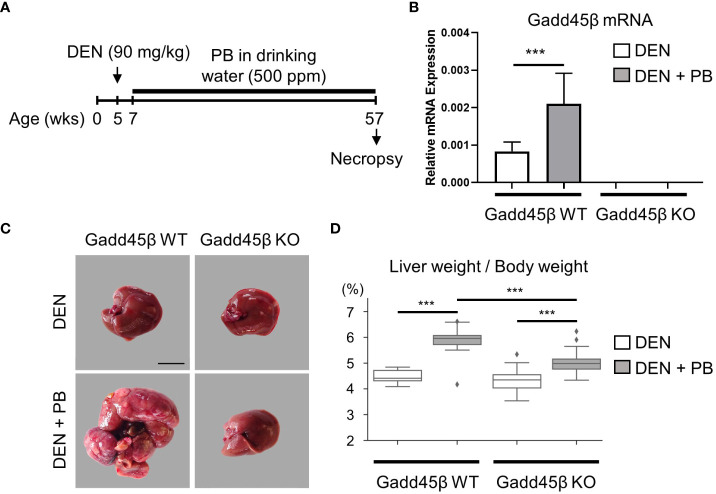
Gadd45β-null mutation compromised HCC promotion by PB. **(A)** Schematic representation of animal treatment. Numbers indicate age of the animals. DEN, diethylnitrosamine; PB, phenobarbital. **(B)** Induction of Gadd45β mRNA by PB in mouse livers. Each value is shown as the mean ± S.D. (n=6). ***, *P* < 0.01 by Student’s *t*-test. **(C)** Carcinoma was developed in DEN/PB-treated wild type mice but not in Gadd45β KO mice. Scale bar = 1 cm. Adenoma and carcinoma development in these mice were summarized in **Table 1** and other liver images of DEN/PB-treated wild type mice were shown in **Supplement Figure 1A**. Representative livers from mice at 50 weeks after PB promotion. The liver from a DEN/PB-treated Gadd45β KO mouse has multiple tumors. Scale bar = 1 cm. **(D)** The liver weight per body weight values in DEN or DEN/PB-treated Gadd45β WT or Gadd45β KO were evaluated. The box plot presents the median, first quartile, and third quartile (n = 16). The vertical bars represent the 1.5*interquartile range (IQR). ***, *P* < 0.001 by one-way ANOVA followed by Sidak’s multiple comparisons.

To evaluate the effect of Gadd45β null mutation for PB-promoted liver tumors in mice, approximately 40 C57BL/6 WT (Gadd45β WT) and 40 Gadd45β null mutant mice (Gadd45β KO) were injected with DEN, and half of the mice in each group were treated with PB in drinking water. As shown in **Figure 2A**, fifty weeks after administration of PB, necropsies were performed, livers were removed, and mRNA expression levels were analyzed (N=6). Real-time PCR confirmed that Gadd45β was not expressed in Gadd45β KO mice (**Figure 2B**). Consistent with our previous results, PB treatment significantly increased the expression of Gadd45β. The numbers of basophilic foci, eosinophilic foci, adenoma, and carcinoma were analyzed in all livers excised from each group. The numbers of foci and tumors in each group are shown in **Table 1**. Five cases of adenoma were observed in Gadd45β WT and only one in Gadd45β KO in the groups with PB treatment (**Table 1**). In addition, carcinoma was observed in only one case in Gadd45β WT, which was not observed in Gadd45β KO (**Table 1**, **Figure 2C**). These results strongly suggested that Gadd45β plays a promoting role in PB-mediated tumorigenesis.

**Table 1:** Incidences of proliferative liver lesions after 50 weeks of PB treatment.

	Treatment	n	Hypertrophy	Basophilic focus	Eosinophilic focus	Adenoma	Carcinoma
*Gadd45β WT*	DEN	17	0/17	0/17	0/17	0/17	0/17
*Gadd45β WT*	DEN+PB	17	16/17(+++)††	0/17	3/17	5/17	1/17†
*Gadd45β KO*	DEN	18	12/18(+ or ++)	1/18	0/18	2/18	0/18
*Gadd45β KO*	DEN+PB	17	17/17(+++)	0/17	6/17	1/17	0/17

†This animal had carcinoma and an adenoma. Adenoma from this animal is included in the total number of hepatocellular adenomas.

††The animal that had the liver with carcinoma was not counted as a sample of hypertrophy.

Subsequently, liver weight was analyzed for the assessment of hepatocyte proliferation. The Grubbs test detected the values of liver weight per body weight of two mice, one with carcinoma and the other with adenoma, of PB/DEN treated Gadd45β WT group as outliers. Therefore, these two samples were excluded in the following analysis (data including those two are shown in **Supplemental Figure 1**). Although liver weight per body weight was increased by PB in both Gadd45β WT and Gadd45β KO mice, that of livers from PB-treated Gadd45β KO mice was significantly smaller compared to that of livers from Gadd45β WT mice (**Figure 2D**). These results support that Gadd45β promotes hepatocyte proliferation in the presence of PB. In contrast, without PB, Gadd45β WT mice developed no adenoma, whereas Gadd45β KO had two adenomas (**Table 1**). This result, which may indicate that Gadd45β functions as a tumor suppressor in the absence of PB, is consistent with previously known Gadd45β function in tumorigenesis.

Eosinophilic foci and adenoma development in rodents are characteristic of liver tumor promotion by PB (32). Eosinophilic foci typically consist of hepatocytes which are stained more eosinophilic and are often larger than surrounding hepatocytes (33.). This is consistent with our previous tumorigenesis study with Car KO mice, which were changed to a liver tumor-susceptible mouse strain by repeated backcrossing to C3H/HeNCrlBR mice. In the present study we also observed PB administration increased eosinophilic foci. However, even though Gadd45β KO mice exhibited lower incidences of adenoma and carcinoma than Gadd45β WT mice in the presence of PB, the same mice showed higher incidences of eosinophilic foci. This histological result indicates that hepatic conditions of eosinophilic foci may not be enough to promote tumorigenesis.

### Gadd45β null mutation causes significant changes in DEN + PB-induced liver gene expressions

**Figure 3:**
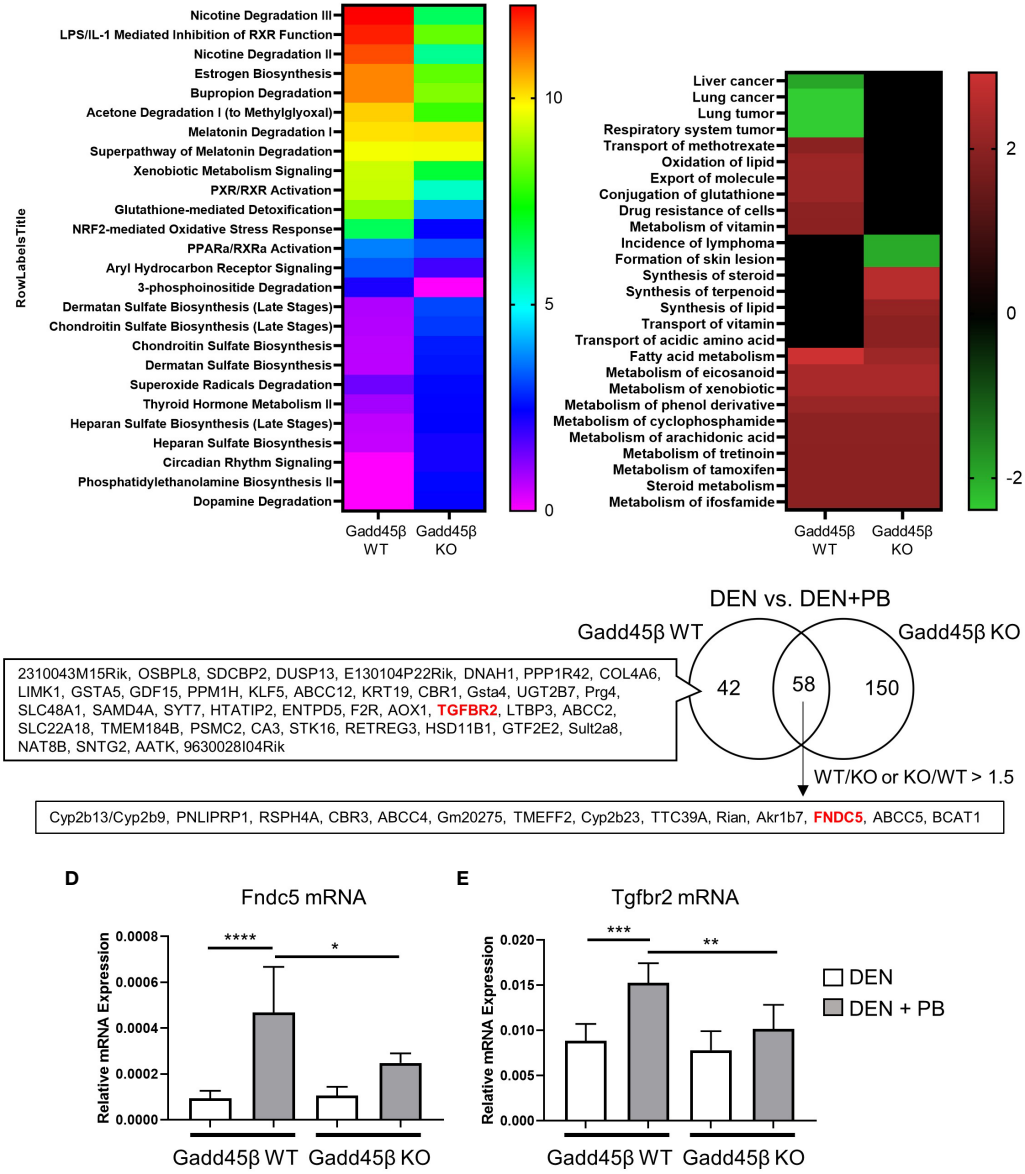
Gadd45β null mutation alters liver transcriptome in DEN + PB treated mice. **(A, B)** Differentially expressed genes of DEN/PB *vs* DEN treated Gadd45β WT or Gadd45β KO mice were analyzed by IPA for Canonical pathways (**A**, -log p value) or Toxicity function (**B**, Z score). **(C)** Venn diagram showing differentially expressed genes in DEN/PB treated *vs* DEN treated Gadd45β WT or Gadd45β KO mice. **(D, E)** Expression levels of Fndc5 mRNA **(D)** or Tgfbr2 mRNA **(E)** in DEN or DEN/PB treated Gadd45β WT or Gadd45β KO mice (n = 6). Data represent the means ± S.D. *P < 0.05, **P < 0.01, ***P < 0.001, or ****P < 0.0001 by one-way ANOVA followed by Sidak’s multiple comparisons.

Results obtained in **Figure 2** suggest that the Gadd45β null mutation provoked fundamental changes in mouse liver responses against DEN/PB treatment. Therefore, we analyzed PB-induced transcriptome responses of wild type and Gadd45β null mutant mice livers expecting to find the key gene expression changes derived from the null mutation resulted in altered tumor development. For this purpose, RNAs isolated from the livers in **Figure 2** were subjected to cDNA microarray analyses as described in the Methods section. We found differentially expressed genes by PB treatment in Gadd45β WT (134 genes) and Gadd45β KO (262 genes) and applied Ingenuity Pathway Analysis (IPA) for the results (**Figures 3A, B**). Canonical pathway analysis results shown in **Figure 3A** suggested Gadd45β gene null mutation provoked widespread effects on PB-induced gene expression in numerous pathways including those involved in endo- and exogenous metabolism. For example, genes in nicotine degradation, estrogen biosynthesis, and melatonin degradation were strongly affected by the null mutation. Furthermore, provocative differences were found by IPA Toxicity functional analysis (**Figure 3B**) in which clear differences were observed in cancer-related genes of multiple tissues including those of the liver. Subsequently, the Venn diagram visualized the unique differentially expressed genes in Gadd45β WT and KO mice (**Figure 3C**, **Supplementary Table 1**). Among those genes, we focused on two genes, *Fndc5* and *Tgfbr2*. Increased FNDC5 expression in human HCC was observed in a previous report (28) and this gene was known to be a CAR target gene (34). On the other hand, *Tgfbr2* was reported to have putative CAR response elements and to be CAR dependently induced in DEN/PB-treated mice (11). Our qPCR confirmed these two genes were induced by DEN/PB compared with DEN alone in WT mice and the inductions were much less prominent in Gadd45β KO mice (**Figures 3D, E**).

### Direct activation of CAR by PB is involved for Fndc5 but not for Tgfbr2 gene inductions

**Figure 4:**
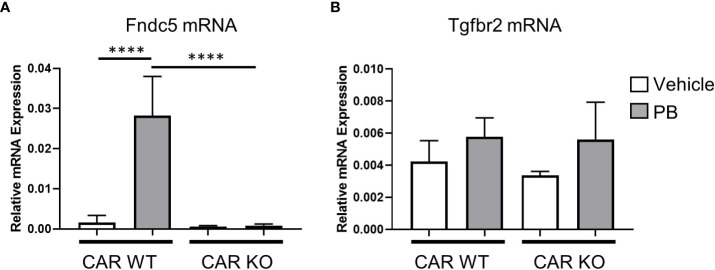
CAR is indispensable for Fndc5 gene induction by PB. Expression levels of Fndc5 mRNA **(A)** or Tgfbr2 mRNA **(B)** in vehicle or PB-treated Car WT or Car KO mice. Data represent means ± S.D. ****, *P* < 0.0001 by one-way ANOVA followed by Sidak’s multiple comparisons.

Next, we assessed the effect of single-dose PB treatment on gene expression levels of *Fndc5* and *Tgfbr2* in CAR WT and KO mice (**Figure 4**). Fndc5 mRNA was induced by PB treatment in CAR WT mice whereas no change was observed in CAR KO mice, indicating *Fndc5* is a CAR target gene. In contrast, *Tgfbr2* was induced by PB treatment in neither CAR WT mice nor CAR KO mice, suggesting the expression change of *Tgfbr2* in DEN/PB-treated Gadd45β WT mice was caused by in-direct functions of CAR activated by long-term treatment with PB.

### Irisin treatment induces proliferation of mouse hepatocyte cell line cells

**Figure 5:**
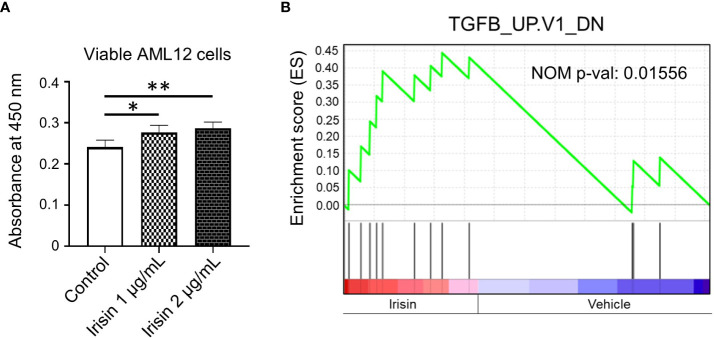
Irisin enhances growth of AML12 mouse liver cells. **(A)** AML12 cells were treated with 1 µg/mL or 2 µg/mL of irisin or vehicle for 72 hr, followed by cell viability determination. Data represent means ± S.D. (n=3). *P < 0.05 and **P < 0.01 by one-way ANOVA followed by Sidak’s multiple comparisons. **(B)** Enrichment plots of genes, downregulated by TGF-β overexpression, in differentially expressed genes by irisin treatment in AML12 cells were presented by GSEA. NOM p-val, nominal *p*-value. The *p*-value was calculated by GSEA.

Irisin has been reported to induce cell proliferations for many cell types including HepG2 cells, one of human hepatocellular carcinoma derived cell lines. Here we analyzed the effects of irisin treatment on the proliferation of mouse hepatocyte AML12. The commercial Irisin we used here has 100% identical peptide sequences between humans and mice. Cell numbers of AML12 cells were increased by irisin treatment after 72 hr, indicating proliferation was promoted by irisin (**Figure 5A**). Irisin induced gene expression changes in AML12 cells were analyzed by cDNA microarray followed by GSEA with C6 gene sets and IPA with upstream analysis (**Figure 5B**, **Table 2**). In this GSEA analysis, we compared Fndc5/Irisin-regulated genes found in our microarray with previously found genes regulated by TGF-β. The comparison revealed genes down-regulated by transiently expression of TGF-β, were enriched in up-regulated genes by irisin treatment (**Figure 5B**). Moreover, the IPA analysis found 10 upstream regulator candidates that are affected by irisin treatment. Among them, irisin has a potential function for suppression of the Tgfbr1 signaling pathway (**Table 2**), suggesting that these factors, Fndc5/Irisin, TGF-β and Tgfbr1, have functional interactions.

**Table 2:** Up-stream analysis for FNDC5 responsive genes in AML12 cells.

Upstream Regulator	Predicted Activation State	Activation z-score	p-value of overlap
RICTOR	Activated	3.198	0.00348
HIRA	Activated	2	0.0112
PHLPP1	Activated	2	0.0275
TGFBR1	Inhibited	-2.111	0.0278
ERF	Inhibited	-2.2	0.00689
DMD	Inhibited	-2.39	0.0134
DSG4	Inhibited	-2.63	0.0014
MYC	Inhibited	-3.003	0.0000148
PLA2G10	Inhibited	-3.13	0.0125
MLXIPL	Inhibited	-3.272	0.00000917

### Irisin/Fndc5 has functional interaction with TGRBR2 for cell proliferation

**Figure 6:**
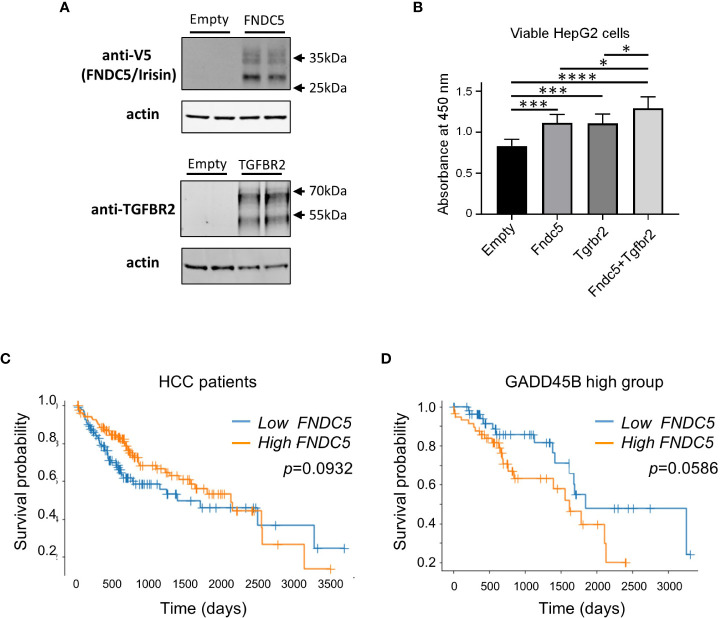
Ectopic expression of Fndc5 and Tgfbr2 enhances HepG2 cell growth. **(A)** FNDC5/Irisin and Tgfbr2 protein expression in HepG2 cells. HepG2 cells were transfected by each expression plasmid and the proteins were detected 72 hr after the transfection by western blotting analyses as described in Methods section. **(B)** HepG2 cells were transfected with mock, Tgfbr2, or Fndc5 expression plasmids and then cultured for 72 hr, followed by cell viability determination. Data represent means ± S.D. (n=3). *P < 0.05, ***P < 0.001, and ****P < 0.0001 by one-way ANOVA followed by Sidak’s multiple comparisons. **(C)** Kaplan-Meier survival curves of patients in the high- (orange) or low- (blue) *FNDC5* expression in the patients are displayed. Differences between survival curves were statistically analyzed by using the log-rank test. **(D)** Kaplan-Meier survival curves of patients in the high- (orange) or low- (blue) *FNDC5* expression in the patients with higher Gadd45β expression are displayed. Differences between survival curves were statistically analyzed by using the log-rank test.

Next, we assessed the effect of irisin treatment on human hepatocarcinoma HepG2 cell growth. Irisin has been reported to bind with Tgfbr2 resulting in interrupted TGF-β1 signaling pathway (35). We assessed the effect of the combination of transiently expressing irisin and TGFBR2 on the proliferation of HepG2 cells. FNDC5/Irisin and Tgfbr2 protein expressions were confirmed by western blotting analyses in **Figure 6A**. The expression of either irisin or TGFBR2 promoted cell proliferation and simultaneous expression of both factors yielded a greater promotion (**Figure 6B**). These results suggested that the two factors have a functional interaction for HepG2 cell proliferation. Next, we analyzed the relation of FNDC5 expression levels with HCC patients’ survival rates (**Figures 6C, D**). Whereas a higher FNDC5 expression group does not show a poorer prognosis (**Figure 6C**), higher FNDC5 expression in higher GADD45B expression groups tends to have a poorer prognosis (**Figure 6D**). Thus, FNDC5 has cooperative effects with GADD45B on prognosis of cancer patients.

## Discussion

Our primary target of this study is defining the molecular mechanism of HCC development in rodent livers in the widely adapted HCC animal model system using DEN as the initiator and PB as the promoter. Here we focused on the roles of Gadd45β in the molecular mechanisms since several lines of evidence suggest possible involvement of this molecule in HCC development. Beyond our expectations, the results in this paper showed that Gadd45β null mutation strongly attenuated HCC development in DEN/PB treated mice (**Figure 2**, **Table 1**). Moreover, in a database analysis we found human survival rates have a strong association with GADD45β expression levels (**Figure 1**), suggesting that Gadd45β is involved in not only HCC of rodents but also that of humans. Although we employed C57BL/6, not a carcinogen-susceptible strain such as C3H, and the number of adenomas and carcinomas observed was not large in this study, the results obtained will be valuable to understand tumorigenesis molecular mechanism in rodent HCC. Nonetheless, the validity of our findings needs to be evaluated in different mouse systems using susceptible strains like C3H. Our genomics analysis using microarray revealed that many genes are affected differently by DEN/PB treatment in wild type compared with Gadd45β null mice. We focused on *Fndc5* and *Tgfbr2* genes among several genes reported to be involved in cell proliferation and apoptosis (**Figure 3**) (27, 36, 37).

**Figure 7:**
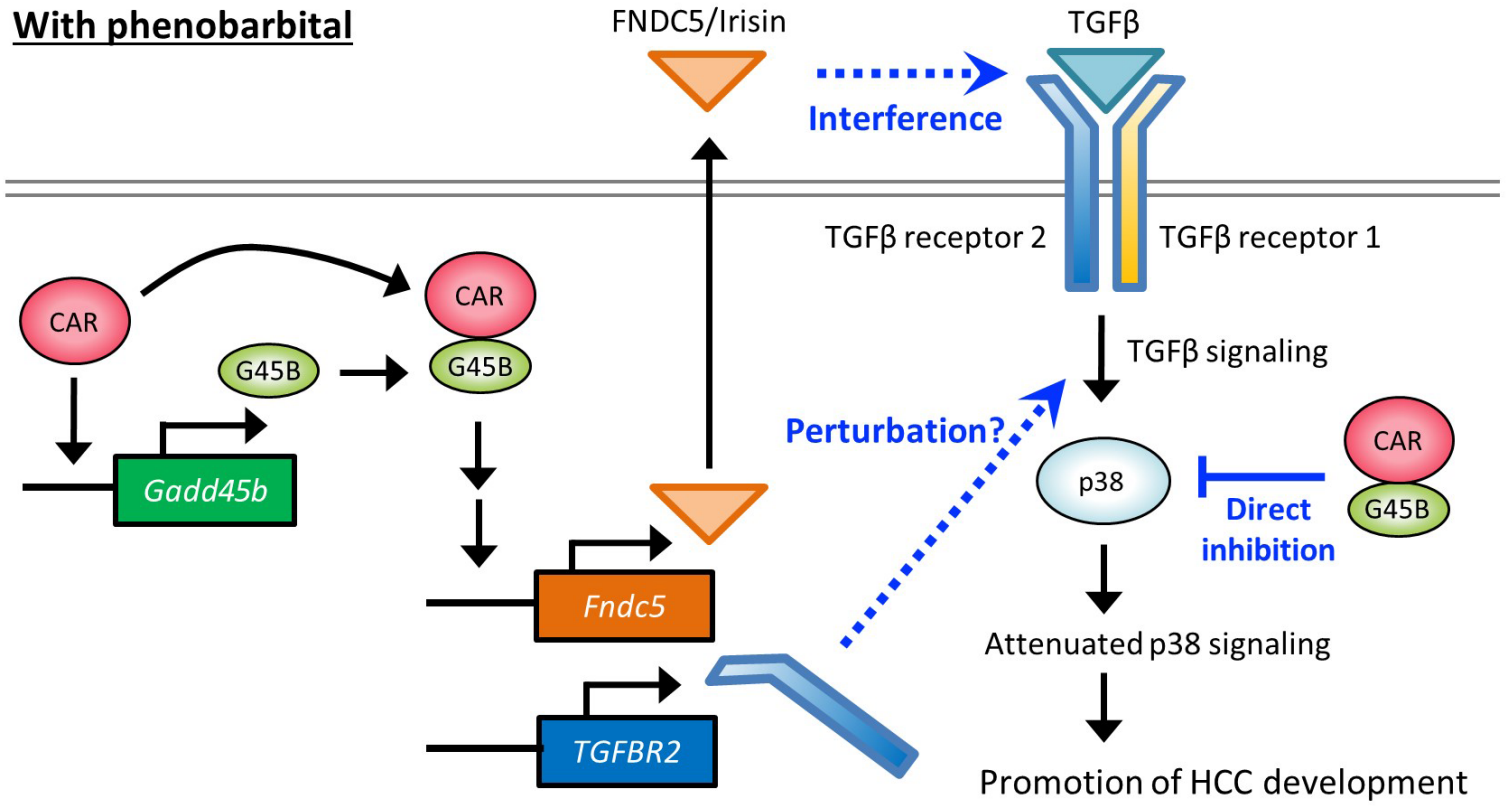
Hypothetical molecular pathways depicting HCC promotion by PB.

Irisin was originally identified as a myokine hormone cleaved from Fndc5 and secreted from muscle cells regulating the browning of white fat tissue (38). Although CAR activation was shown to induce Fndc5 mRNA expression in the liver (34), the molecular and physiological roles of Fndc5 in this organ have not been well understood (27, 37). Recent studies have shown that irisin promotes the proliferation of osteoblasts (39), endothelial cells (40), neural cells (41), and pancreatic β cells (42) but suppresses the proliferation of lung cancer cells (43). In this study, we demonstrated that the growth of mouse AML12 cells and human HepG2 cells are induced by irisin treatment. Irisin binds to the TGF receptor complex that is composed of the TGFBR2 dimer (35) and may disrupt the TGF signaling pathway and affect cell growth (44, 45) (**Figure 7**). Indeed, cDNA microarray analysis of AML12 cells suggested an intimate relation of irisin and TGFBR2. Gene expression changes induced by irisin in AML12 cells are negatively associated with genes under the regulation of TGFBR1 in IPA analysis (**Table 2**). TGFBR2 is playing a major role, forming a heterotetramer with TGFBR1 or forming homotetramer, in the TGF-β signaling pathway (36, 46). This means a substantial part of the genes under TGFBR1 regulation found in **Table 2** are also regulated as downstream genes of TGFBR2. Therefore, the results in **Table 2** are implying that irisin and TGFBR2 are functionally interacting in AML12 cells treated with irisin.

**Figure 7** proposes hypothetical molecular pathways involved in HCC promotion by PB. Our previous reports using mice treated with PB for long or short periods and cell-based assays have indicated that PB increased CAR-Gadd45β complex that suppresses anti-tumor p38 MAPK activity. The present study with Gadd45β KO mice demonstrated that Gadd45β is essential to the PB-mediated full development of hepatic adenoma and HCC as well as irisin/Fndc5 and Tgfbr2 up-regulation by PB. Irisin can interfere with the TGF-β signaling pathway through the direct interaction with the receptor complex and the induction of Tgfbr2 may perturb this pathway by changing the receptor complex stoichiometry. Overall, our data implies that repression of the p38 MAPK through interference with the TGF-β pathway by the two molecules contributes to HCC development promotion. However, evidence at this point is not enough to describe the details of the molecular functions of these proteins in this pathway, and further studies are necessary for deciphering molecular-level mechanisms that depict how these molecules are involved in PB promoted HCC development in rodents.

In rodents, various non-genotoxic substances, including PB, induce liver carcinogenesis through the mode of action involving CAR activation. However, this phenomenon has not been established in humans (47), and the molecular mechanism depicted in **Figure 7** may not have full legitimacy in humans. Nevertheless, even without the direct involvement of CAR, partially overlapped signaling pathways may be encompassed in human HCC development. In fact, there are reports of elevated expression of FNDC5/irisin in human hepatocellular carcinoma (HCC) (28) and our bioinformatic analysis also indicates that high expression of both GADD45β and FNDC5 negatively impacts the prognosis of cancer patients (**Figure 6D**). Thus, the elucidation of the detailed molecular mechanistic roles played by GADD45β and FNDC5 in liver cancer will contribute to finding new therapeutic targets. On the other hand, multiple studies suggest associations of human liver carcinogenesis with decreased expression of TGFBR2, which appears to contradict our findings in mice (48, 49) (**Figure 2C**, **Figure 3E**). Therefore, TGF-β pathways disruption in rodents arises from an imbalance of receptor subunits caused by augmented TGFBR2 expression may not happen in humans for HCC development in the same fashion. This difference may be related to the difference between humans and rodents regarding PB-induced HCC mode of action.

Recently, the Yes-associated protein (YAP) signaling in the Hippo pathway is reported to be an accelerator of hepatocyte proliferation by CAR activation. The Hippo pathway regulates organ size in mammals (50), and functioning as a downstream effector, YAP is involved in the development of HCC (50–53). YAP has been shown to facilitate CAR-mediated hepatocyte proliferation (54–56). TCPOBOP-induced activation of CAR increased the accumulation of YAP in the nucleus and enhanced cell proliferation (57). The functional interaction between TGFBR2 and YAP has been reported in both humans and mice (58, 59), implying that the CAR-YAP pathway could affect TGFBR2 molecular roles proposed in **Figure 7** for the CAR-dependent proliferation of hepatocytes. In addition, recent findings suggest that CAR suppresses hepatocellular carcinoma through the erythropoietin signaling pathway in humans (60). Exploring the relationship between YAP pathway or erythropoietin pathway and the pathway depicted in **Figure 7** would be a crucial research objective in the future.

In conclusion, the results of this study demonstrate for the first time, to our knowledge, that Gadd45β is a factor in promoting PB-mediated tumorigenesis in mice. In addition, we identified irisin as a possible curial player for HCC development in mice and humans. These findings shed more light on the biological process underlying HCC development.

## Data availability statement

GEO accession numbers for microarray study results in this report are GSE232188 and GSE231793. The data will be released to the public on May 10, 2024.

## Ethics statement

The animal study was approved by Animal Care and Use Committee, NIEHS, NIH. The study was conducted in accordance with the local legislation and institutional requirements.

## Author contributions

TH, MN, KY, and TS contributed to conception and design of the study. TH and RM collected animal study data. KY and TS performed genomic data analyses. TH and KY performed the statistical analysis. TH, KY, MN, and TS wrote the first draft of the manuscript. All authors contributed to manuscript revision, read, and approved the submitted version. 

## Supplementary material

Supplementary Figure 1 Effects of DEN/PB on liver and body weight in Gadd45β WT and KO. **(A)** Liver images of WT DEN/PB treated mice. Adenomas were found in livers shown in panels 1 – 3. No tumor was observed in the livers in panels 4 – 8. The liver weight **(B)**, body weight **(C)**, and the liver weight per body weight values **(D)** in DEN or DEN/PB-treated Gadd45β WT or Gadd45β KO were evaluated. The box plot presents the median, first quartile, and third quartile. The vertical bars represent the 1.5*interquartile range (IQR). **P* < 0.05 and ***P* < 0.01 by one-way ANOVA followed by Sidak’s multiple comparisons.

Supplementary Figure 2 Effects of Gadd45β on the expression of Cyp2b10 mRNA, Cyp2b9 mRNA, and Cyp2b13 mRNA. Expression of Cyp1b10 mRNA **(A)**, Cyp2b9 mRNA **(B)**, and Cyp2b13 mRNA **(C)** were analyzed in Cadd45β WT and KO mice. **(D)** Expression of Cyp2b10 mRNA was analyzed in CAR WT and KO mice. **P* < 0.05, ***P* < 0.01, and ****P* < 0.001 by one-way ANOVA followed by Sidak’s multiple comparisons.

Supplementary Figure 3 Effects of Gadd45β on the expression of multiple genes. Relative mRNA expression was analyzed by real-time PCR for liver samples obtained from Gadd45β WT or KO mice. **P* < 0.05, ***P* < 0.01, and ****P* < 0.001 by one-way ANOVA followed by Sidak’s multiple comparisons.

Supplementary Table 1 Differentially expressed genes in DEN/PB treated *vs* DEN treated Gadd45β WT or Gadd45β KO mice. **(A)** Differentially expressed genes by PB/DEN treatment compared with DEN treatment in Gadd45β WT mice. **(B)** Genes grouped in the common group in **Figure 3C**, differentially expressed at least 1.5-fold between Gaddd45β WT and Gadd45β KO mice.
