# Supplementary figures and images for: CAR requires Gadd45β to promote phenobarbital-induced mouse liver tumors in early stage
Source: Front Oncol. 2023 Sep 7;13:1217847. doi: 10.3389/fonc.2023.1217847 (PMC10516603; doi:10.3389/fonc.2023.1217847)

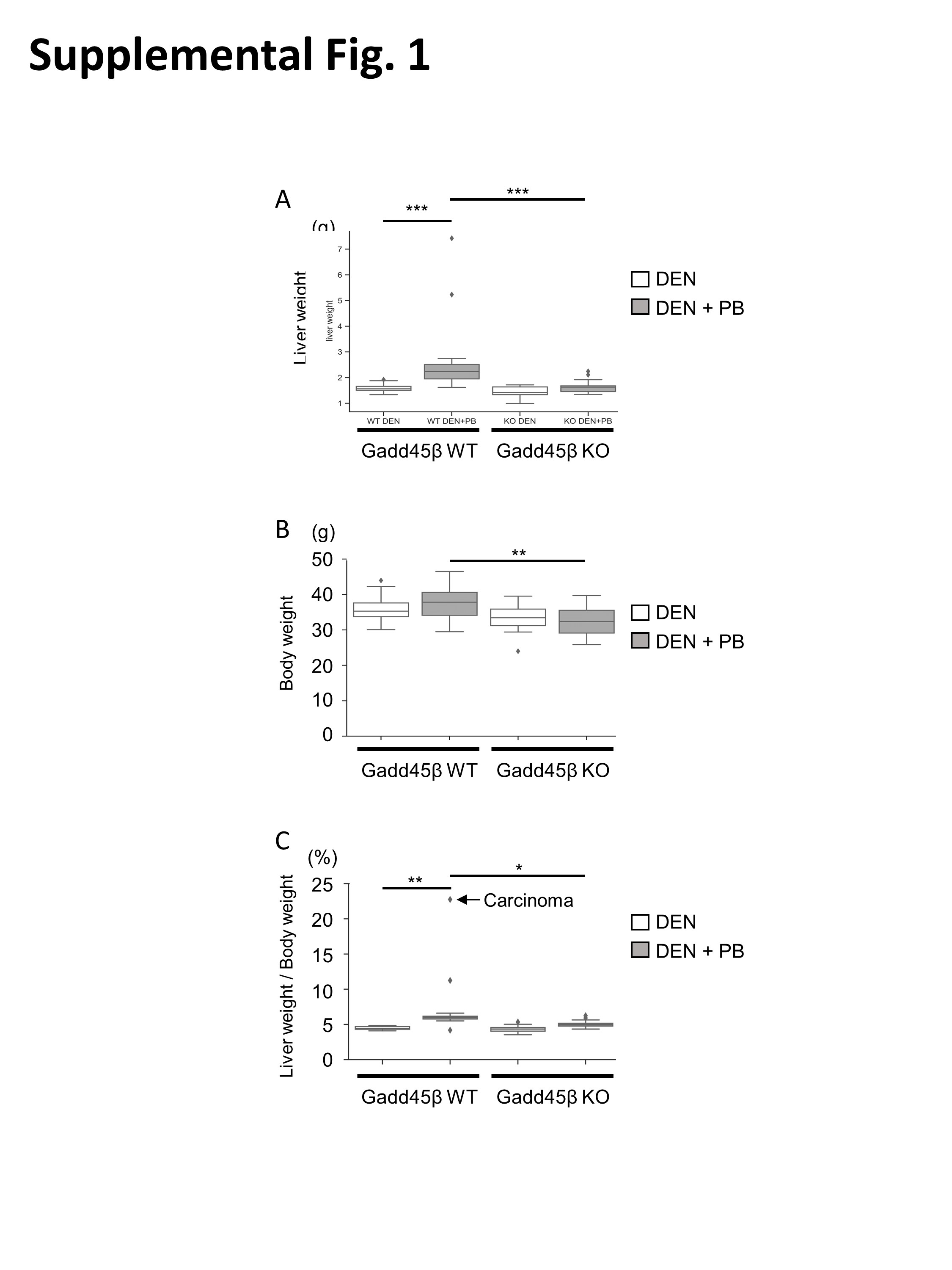

Supplement: Supplementary Figure 1 — Effects of DEN/PB on liver and body weight in Gadd45β WT and KO. (A) Liver images of WT DEN/PB treated mice. Adenomas were found in livers shown in panels 1 – 3. No tumor was observed in the livers in panels 4 – 8. The liver weight (B), body weight (C), and the liver weight per body weight values (D) in DEN or DEN/PB-treated Gadd45β WT or Gadd45β KO were evaluated. The box plot presents the median, first quartile, and third quartile. The vertical bars represent the 1.5*interquartile range (IQR). *P < 0.05 and **P < 0.01 by one-way ANOVA followed by Sidak’s multiple comparisons. [file Image_1.jpg]

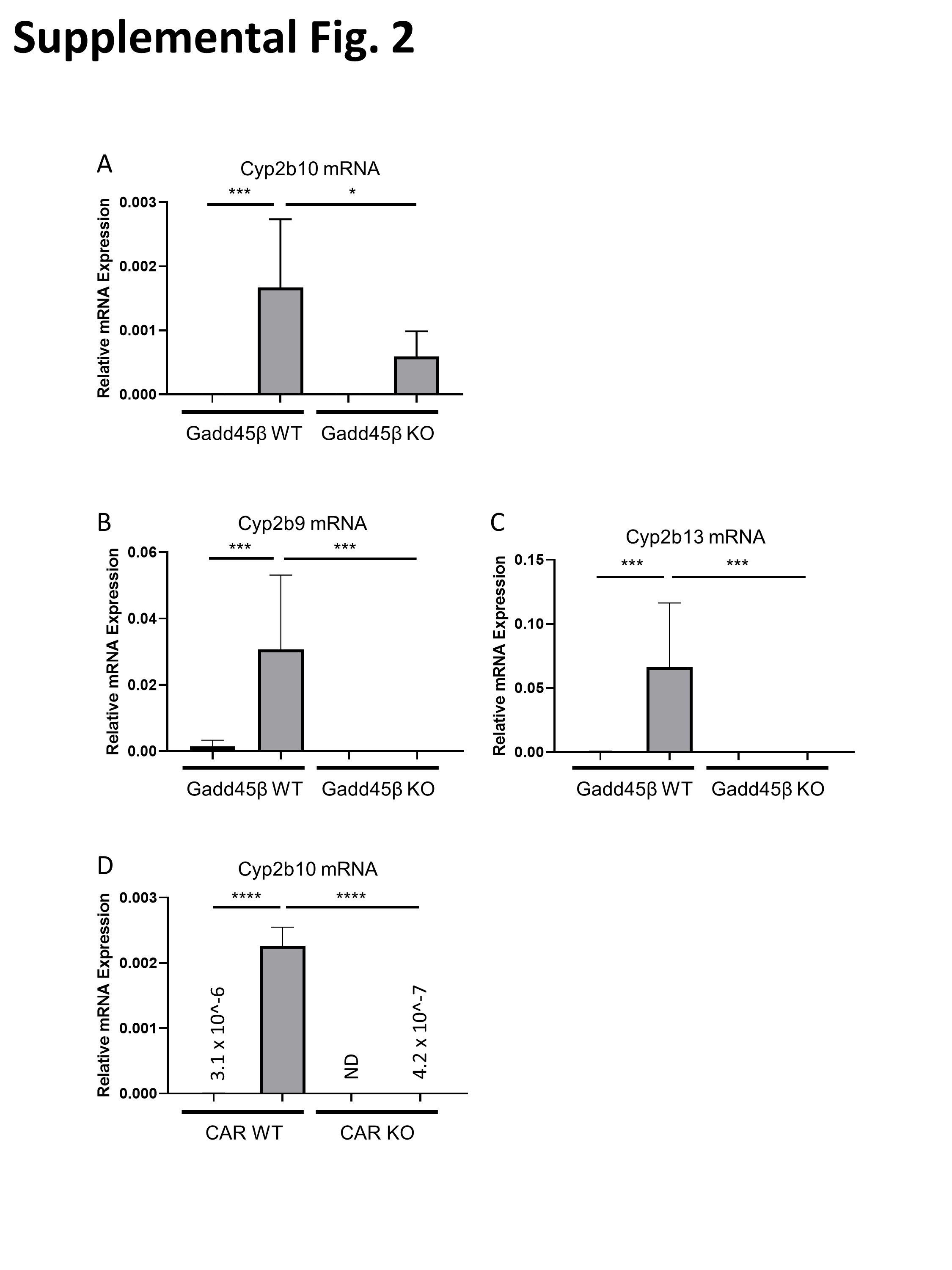

Supplement: Supplementary Figure 2 — Effects of Gadd45β on the expression of Cyp2b10 mRNA, Cyp2b9 mRNA, and Cyp2b13 mRNA. Expression of Cyp1b10 mRNA (A), Cyp2b9 mRNA (B), and Cyp2b13 mRNA (C) were analyzed in Cadd45β WT and KO mice. (D) Expression of Cyp2b10 mRNA was analyzed in CAR WT and KO mice. *P < 0.05, **P < 0.01, and ***P < 0.001 by one-way ANOVA followed by Sidak’s multiple comparisons. [file Image_2.jpg]

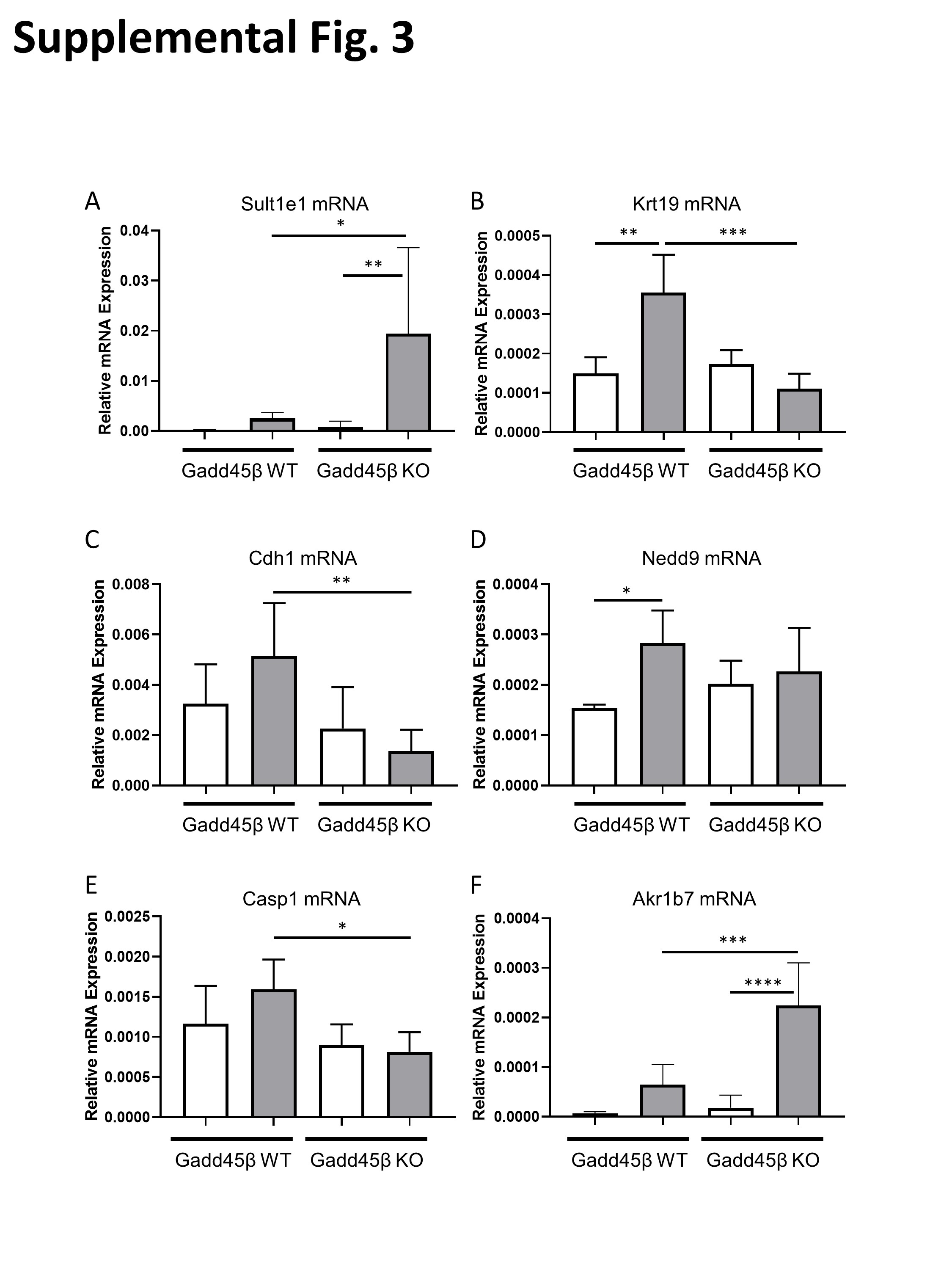

Supplement: Supplementary Figure 3 — Effects of Gadd45β on the expression of multiple genes. Relative mRNA expression was analyzed by real-time PCR for liver samples obtained from Gadd45β WT or KO mice. *P < 0.05, **P < 0.01, and ***P < 0.001 by one-way ANOVA followed by Sidak’s multiple comparisons. [file Image_3.jpg]
