# Supplementary material for: CAR requires Gadd45β to promote phenobarbital-induced mouse liver tumors in early stage
Source: Front Oncol. 2023 Sep 7;13:1217847. doi: 10.3389/fonc.2023.1217847 (PMC10516603; doi:10.3389/fonc.2023.1217847)
Supplement: Supplementary Table 1 — Differentially expressed genes in DEN/PB treated vs DEN treated Gadd45β WT or Gadd45β KO mice. (A) Differentially expressed genes by PB/DEN treatment compared with DEN treatment in Gadd45β WT mice. (B) Genes grouped in the common group in Figure 3C , differentially expressed at least 1.5-fold between Gaddd45β WT and Gadd45β KO mice. [file Table_1.pdf]

Supplemental Table 1

A

Symbol	Expr Fold Change(A1)	Expr p-value(A1)
2310043M15Rik	8.741	0.0442
OSBPL8	7.609	0.0399
SDCBP2	6.82	0.0157
DUSP13	6.236	0.0198
E130104P22Rik	5.462	0.0285
DNAH1	5.117	0.0309
PPP1R42	4.77	0.0318
COL4A6	3.608	0.03
LIMK1	3.234	0.041
GSTA5	3.026	0.0347
GDF15	2.771	0.0286
PPM1H	2.675	0.0495
KLF5	2.621	0.0273
ABCC12	2.403	0.021
KRT19	2.303	0.0145
CBR1	2.16	0.0103
Gsta4	1.997	0.041
UGT2B7	1.996	0.0325
Prg4	1.944	0.0084
SLC48A1	1.915	0.0156
SAMD4A	1.905	0.0033
SYT7	1.835	0.0495
HTATIP2	1.793	0.0239
ENTPD5	1.763	0.0367
F2R	1.702	0.0285
AOX1	1.653	0.0198
TGFBR2	1.635	0.0203
LTBP3	1.526	0.0166
ABCC2	1.492	0.0183
SLC22A18	1.487	0.0289
TMEM184B	1.448	0.0245
PSMC2	1.241	0.0392
CA3	-1.242	0.0066
STK16	-1.351	0.039
RETREG3	-1.367	0.0286
HSD11B1	-1.408	0.0414
GTF2E2	-1.428	0.0452
Sult2a8	-1.466	0.0405
NAT8B	-1.529	0.0348
SNTG2	-1.907	0.03
AATK	-3.476	0.0298
9630028I04Rik	-14.204	0.0003

B

Symbol	Expr Fold Change(A1)	Expr p-value(A1)	Expr Fold Change(A2)	Expr p-value(A2)
Cyp2b13/Cyp2b9	84.541	0.0005	131.026	0.0006
PNLIPRP1	37.002	0.0002	73.73	0.0000897
RSPH4A	20.051	0.0044	7.481	0.0401
CBR3	16.643	0.0012	7.508	0.0104
ABCC4	13.776	0.005	8.03	0.02
Gm20275	13.337	0.004	5.434	0.0401
TMEFF2	12.342	0.0318	40.705	0.0297
Cyp2b23	9.562	0.001	5.814	0.0048
TTC39A	7.837	0.0016	4.132	0.019
Rian	7.285	0.0407	33.434	0.0009
Akr1b7	6.946	0.0002	14.846	0.0000286
FNDC5	3.876	0.004	2.527	0.0359
ABCC5	3.751	0.0045	1.551	0.0342
BCAT1	-12.494	0.0002	-4.912	0.0049
